# Phylogenomics Analysis of SARS-CoV2 Genomes Reveals Distinct Selection Pressure on Different Viral Strains

**DOI:** 10.1155/2020/5746461

**Published:** 2020-11-27

**Authors:** Sanjana Ghosh, Sandipan Chakraborty

**Affiliations:** Amity Institute of Biotechnology, Amity University, Kolkata 700135, India

## Abstract

We are witnessing a tremendous outbreak of a novel coronavirus (SARS-CoV2) across the globe. Upon exposure to different population and changing environment, the viral strain might experience different mutational bias that leads to genetic diversity among the viral population. Also, the diversification can be influenced by distinct selection pressure on different viral genomes. We have carried out a comparative genomic analysis of 82 SARS-CoV2 genomes. We have evaluated their evolutionary divergence, substitution pattern, and rates. Viral genomes under distinct selection pressure have been identified. Sites that experience strong selection pressure also have been identified. Our result shows that the translational preference of a few codons is strongly correlated with the mutational bias imposed by genome compositional constraint and influenced by natural selection. Few genomes are evolving with a higher mutational rate with a distinct signature of nucleotide substitution in comparison to others. Four viral strains are under the effect of purifying selection, while nine SARS-CoV2 genomes are under strong positive selection bias. Site analysis indicates a strong positive selection pressure on two codon positions at 3606th and 8439th positions. Our study elucidates adaptation of few SARS-CoV2 viral strain during the outbreak shaping by natural selection and genomic compositional constraints.

## 1. Introduction

Recent pandemic of a new coronavirus, SARS-CoV2, infects more than 40 million people and we have witnessed 1 million deaths already. Genomic comparison of SARS-CoV2 from infected individual shows 79.6% sequence identity to SARS-CoV and 96% identity to bat coronavirus, BatCoV RaTG13, which suggests a possible bat origin of the new virus [1]. Genome comparison reveals the possible role of pangolin as an intermediate host during the cross-species transfer of the virus [2], although it is still highly debatable [3, 4]. SARS-CoV2 genome is a positive single-stranded RNA of ~30 kb size, comprises of a 5'-UTR, a long ORF1a/b codes different nonstructural proteins, *S* gene, which encodes spike glycoprotein, other genes that encode for the envelope E protein and the membrane protein M, region encoding the nucleocapsid N protein followed by 3'-UTR [5]. Data available from the World Health Organization (WHO) reveals that the rate of infectivity and associated morbidity reported in different countries is different. The United States of America and Europe are severely affected by reported cases of more than 18 million and 8 million, respectively. Eastern Mediterranean regions reported 2 million cases of SARS-CoV2 infection while South-East Asia and Western Pacific regions reported cases are ~8.5 million and ~0.7 million, respectively. Surprisingly, there is only ~1.2 million reported cases of SARS-CoV2 from Africa. Also, data on the rate of infectivity of the virus increased severely during the pandemic. The origin of such increased virulence can be hidden in the evolution of SARS-CoV2 during the pandemic. However, very little is known about the genetic variability of this novel virus across the different population. Upon exposure to different population and changing environment, the virus populations might experience different selection pressure that leads to several closely related genomic variants [6, 7]. Although the timescale of the pandemic is short, the mutation rates and rapid replicative kinetics of the viruses may lead to diverse SARS-CoV2 genomes across the whole population. Noteworthy, the RNA virus shows a high degree of adaptive potential [8]. Thus, it is important to assess the genomic diversity of SARS-CoV2 owing to the high mutation rate of RNA viruses. Genetic diversification is a dynamic and complex process. Therefore, the viral genome in the different population may be under different selection pressure, purifying/positive or neutral. Thus, it is important to access the selection pressure of different micropopulation of viral genomes to understand the origin of genetic diversification.

Here, we have carried out a detailed comprehensive analysis of the coding region of 82 SARS-CoV2 genomes from diverse population across the world to understand their acquired genetic diversity. We have evaluated their evolutionary divergence, substitution pattern, and rates. Also, we have identified the viral genomes among the population which is under evolutionary selection pressure. The sites of the genome that experience particular selection pressure across the micropopulations also have been identified.

## 2. Materials and Methods

### 2.1. Data Collection

NCBI Genome database contains full-length genomic sequences of 92 SARS-CoV2 viral strains from the human host across the world. Analysis of the annotation of genome and initial alignment revealed 10 genomes with the missing region. Those genomes were discarded from further analysis. The final set retained 82 viral genomes and the details of the genomes are shown in Table S1 (supporting information). Each genome was manually checked to identify the CDS region.

### 2.2. Sequence Analysis

The whole-genome alignment of 82 SARS-CoV2 genomes was performed with Clustal Omega [9]. Aligned sequences were imported in MAGA-X [10] version 10.1 and analysed for several genomic composition analysis on the aligned genomes and its corresponding aligned translated proteins.

### 2.3. Evolutionary Analysis

Statistical quantities like nucleotide frequencies, codon usage pattern, and transition/transverse rate were calculated using MEGA-X. Sequences were then analysed for the identification of the best-suited nucleotide substitution model by performing Maximum Likelihood fits on 24 different substitution models. The Tamura-Nei model with nonuniformity of evolutionary rates among sites modelled by using a discrete Gamma distribution (+G) with 5 rate categories was found to be the best-suited one, judged by the lowest Bayesian Information Criterion score. The estimated gamma shape parameter was found to be 0.05. Estimation of evolutionary distance between sequences was calculated by calculating the number of base substitutions per site between sequences using the maximum composite likelihood model considering the rate variation [11]. Pattern disparities between sequences were estimated by calculating the homogeneity in the substitution patterns [12].

### 2.4. Phylogenetic Analyses

The phylogenetic tree was constructed by using MEGA-X [10]. The tree was constructed using the maximum likelihood (ML) method. The Tamura-Nei nucleotide substitution model with a discrete Gamma distribution of five different rate categories (shape parameter = 0.05) was used. Bootstrap resampling of 500 times was performed to obtain the most likely phylogenetic tree.

### 2.5. Selective Pressure Analysis

A codon-based selection pressure analysis was performed to identify purifying selection (dN < dS) and positive selection (dN > dS) on the CDS of 82 genomes using the Nei-Gojobori method [13] implemented in MEGA-X. Genomes with negative and positive selection pressure, judged by the *p* values, were considered separately to identify site-wise selection pressure throughout the genome using the Datamonkey web-server (https://www.datamonkey.org/). Genomes with evidence of purifying and positive selection were aligned separately to obtain in-frame codon alignment using MAFFT, and then the phylogenetic trees were reconstituted using the maximum likelihood method. The aligned sequences with the reconstituted phylogenetic trees were then analysed with the SLAC (single likelihood ancestor counting) method to reconstruct the ancestral state using the maximum likelihood method [14].

## 3. Result and Discussions

### 3.1. Compositional Characterization of SARS-CoV2 Genomes

**Figure 1:**
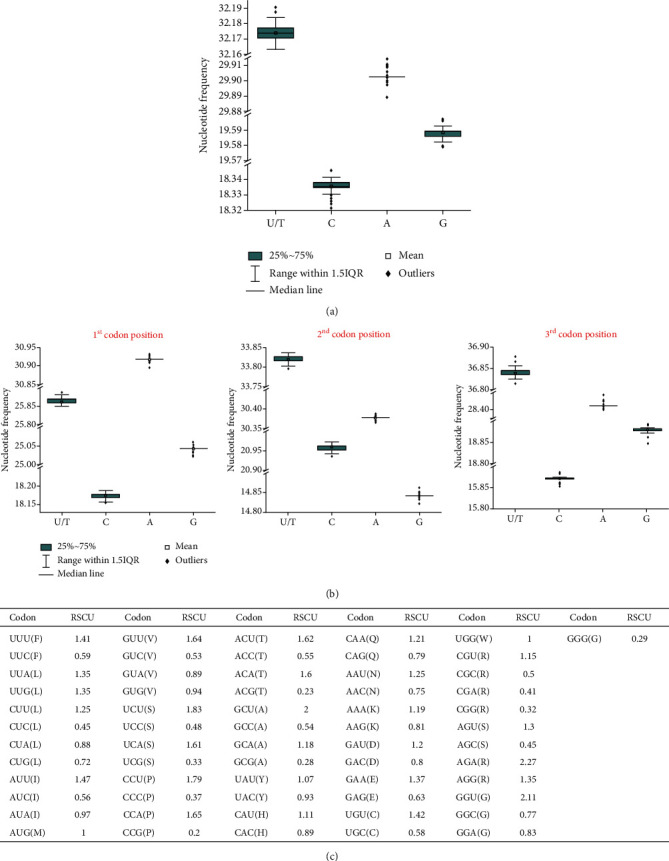
Distribution of nucleotide frequency in all the 82 SARS-CoV2 genomes (a) and at each of the three codon positions in all the genomes are shown (b). (c) Codon usage pattern along with the relative synonymous codon usage (RSCU) values for each codon is listed.

SARS-CoV2 genomes are found to be AT/U-rich, as observed in other related RNA viruses. The average AT/U content is 62.07 ± 0.004% (Figure 1(a)). However, great diversity in AT/U content has been observed at each codon position. At the first codon site, the average value decreases to 56.78% which is primarily due to the lower occurrence of T/U and the higher prevalence of G at that particular site (Figure 1(b)), in comparison to the average. According to the codon usage pattern, the alteration leads to nonsynonymous substitution between valine and phenylalanine in the population (Figure 1(c)). Relative synonymous codon usage (RSCU) values indicate higher usage of both GUU(V) and UUU(F) codon in the SARS-CoV2 population (Figure 1(c)). However, the 2nd and 3rd codon positions are more biased towards the AT/U content with an average value of 64.19% and 65.24%, respectively. Although the GC-content is lower in those sites in comparison to the average, at the 2nd codon position, the prevalence of G content is significantly lower. Evident from the codon usage pattern (Figure 1(c)), only six codons with G at the 2nd codon site exhibit RSCU values > 1. These are UGU(C), CGU(R), AGU(S), AGA(R), AGG(R), and GGU(G). At the 3rd codon position, T/U is abundant in the genome, whereas C-content is lowest. Notably, no codon with C at the 3rd site is frequently used to encode viral proteins, RSCU < 1.

Genomes of (-)-RNA viruses are more AT-rich than (+)-RNA. Particularly, compositional bias towards high A-content was previously reported in the coding strands for (-)-RNA [15]. Among the (+)-RNA viruses, the GC-content of SARS coronaviruses are on the lower side of the GC-content scale [15]. Thus, the mutational bias greatly differs in RNA viruses with different genome polarity due to different mutational pressure. Previous reports on human coronaviruses show great variance in the nucleotide composition among different genomes. In general, human coronaviruses are AT/U-rich and C-content is particularly low. Among them, MERS appears to be associated with the highest C-content (20.3%), while the lowest C-content has been reported for HKU (12.9%). C-content of SARS-CoV2 is a little lower than SARS and MARS, but considerably higher than other coronaviruses, like HKU isolates, OC43, and NL63 [16]. Variation in the A-content is very less significant among different coronavirus species. However, U/T content varies greatly among coronaviruses. The highest U content is reported in isolates of HKU, while SARS-CoV2 and SARS are on the lower side of the scale [16]. Thus genomic content varies greatly even in related coronaviruses, which indicates different mutational biases on different coronavirus genomes.

Codon usage pattern also reveals more frequent utilization of codons with U at the first codon site. For example, leucine is encoded by six codons; among them, two codons, UUA and UUG, are more frequently utilized with RSCU values of 1.35. Among the rest of the four codons with C at the first site, CUU is associated with RSCU values of 1.25, while the other three codons are less frequently used (RSCU values ≪ 1). It has been often observed that both translational selection and compositional constraints dictate the codon utilization variation. Overall, we have observed that the compositional constraints mostly dictate the translational biases for SARS-CoV2 genomes. Apparent from the RSCU values of the codons in different SARS-CoV2, genomes show the clear translational preference of few codons. Notably, selection pressure also induces the nonuniformity of codon usage under the influence of compositional bias.

### 3.2. Evolutionary Distances and Pattern Disparity in SARS-CoV2 Genomes

We have identified 119 variable regions in 82 SARS-CoV2 genomes. Among them, 28 are the parsimony-informative sites where at least two different nucleotides appear at least in a frequency of two. These changes lead to 79 variable sites in the translated proteins; among them, 17 are the parsimony-informative sites. We have further assessed the evolutionary divergence in 82 genomes by calculating the number of base substitutions per site between a pair of genomes using maximum composite likelihood model, where the rate variation among sites was modelled using a gamma distribution with the shape parameter of 0.05.

**Figure 2:**
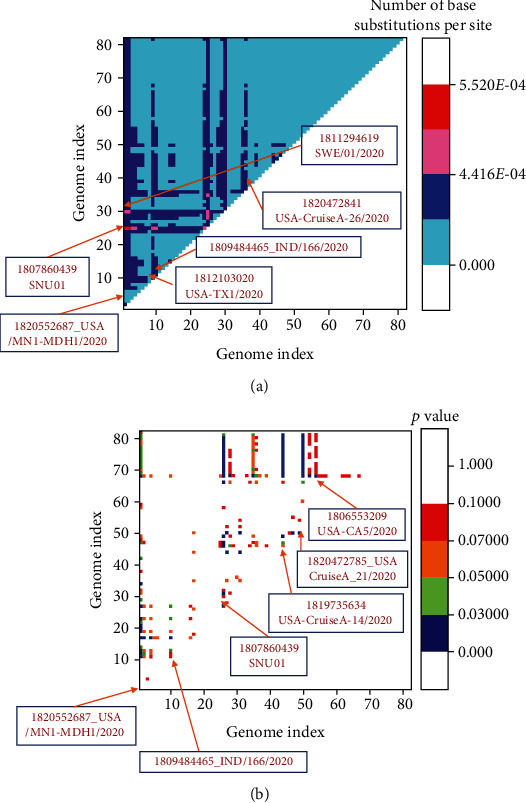
(a) Evolutionary divergence of 82 genomes is shown. Divergence is shown in terms of the number of base substitutions per site between a pair of genomes. (b) Homogeneity in the substitution patterns between the 82 genomes is shown. The probability of rejecting the null hypothesis, i.e., sequences evolved with the same pattern of substitution, is represented.

Results are summarized in Figure 2(a). Six genomes show distinct evolutionary divergences from the genome pool. One SARS-CoV2 genome from South Korea, three genomes from three different states of the USA, one genome from India, and one from Sweden evolve with a higher rate of substitution in comparison to the other genomes. These indicate that these genomes under definitive selection pressure and are trying to adapt. We have further categorised all the genome based on their pattern disparity index. We have tested the homogeneity in the substitution patterns between the 82 genomes. The probability of rejecting the null hypothesis, i.e., sequences evolved with the same pattern of substitution, judged by the disparity index is shown in Figure 2(b). Interestingly, the identified genomes which displayed evolutionary divergence from the other SARS-CoV2 genomes also display pattern disparity. Those genomes are not only evolving with a higher rate but also with a different pattern of mutations.

### 3.3. Phylogenetic Analysis

**Figure 3:**
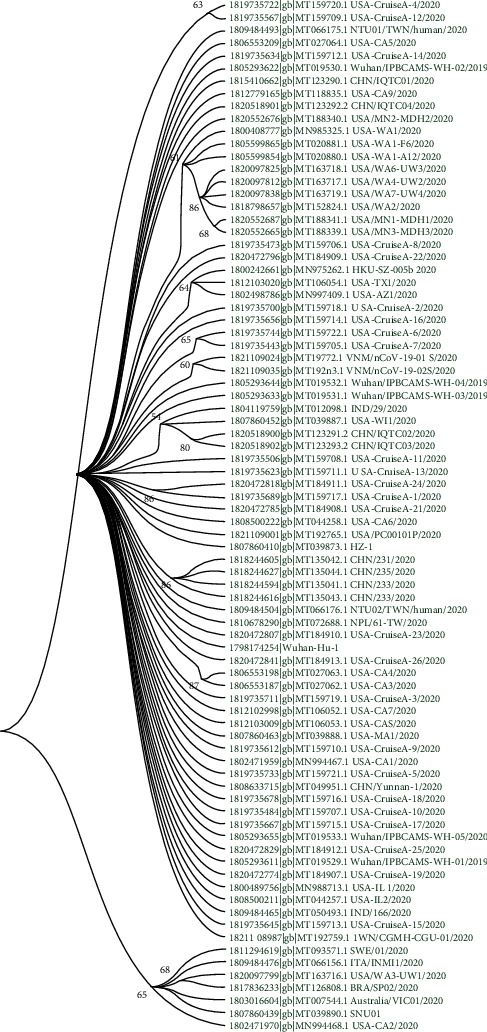
Maximum likelihood phylogenetic tree of 82 SARS-CoV2 genomes from different demographic regions of the world constructed by using MEGA-X.

We have further analysed the genetic diversity of SARS-CoV2 genomes during the pandemic using phylogenetic analysis using the maximum likelihood method.  Figure 3 represents the maximum likelihood tree of SARS-CoV2 genomes. Genetic distances among genomes are small which is rational because the novel virus emerged very recently and the timescale of evolution is very small. Thus, most of the viral genomes are very closely related and belong to a primary clade. Interestingly, genomic sequences of SARS-CoV2 reported in the same state of USA clubbed together, but among the states, they are differently clubbed.

Viral genomes from the USA are often closely clubbed with the viral genomes from China. Notably, the first SARS-CoV2 outbreak was reported in Wuhan, China. Thus, the Chinese viral strain is the probable ancestral origin of the SARS-CoV2 genomes reported from the USA. Interestingly, two Indian viral genomes from the same state are clubbed differently suggesting different ancestral origins. Genome with accession number MT012098.1 clubbed together with genomes from USA and China while the other genome (accession number: MT050493.1) is closely related to the genome from Taiwan, China (accession number: MT192759.1).

Noteworthy, viral genomes from Brazil, Australia, South Korea, Sweden, and Italy are clubbed together in a distinct subclade with a viral genome from California, USA. This clade does not contain any viral genome from Chinese origin which indicates that this group of SARS-CoV2 virus is evolved during the later stages of a pandemic. These genomes are characterized by high and distinct mutational biases thus possibly evolving with selection pressure.

### 3.4. Analysing Evolutionary Pressures in SARS-CoV2 Genomes

**Figure 4:**
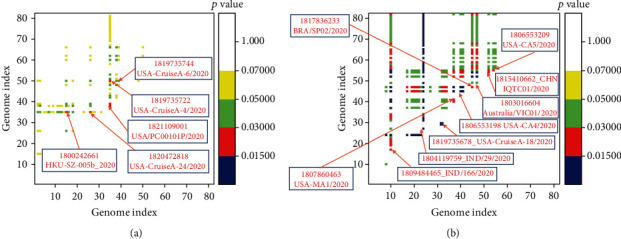
Identification of SARS-CoV2 genomes under purifying selection bias (a) and positive selection bias (b) by using the codon-based test aided by the Nei-Gojobori method is shown. The probability of rejecting the null hypothesis of strict-neutrality (dN = dS) in favour of the alternative hypothesis (dN < dS for purifying selection and dN > dS for positive selection) is represented as shown in different colours.

Our analysis clearly shows that the genetic variations in SARS-CoV2 genomes are not evenly distributed. We have therefore analysed all the 82 genomes to identify those genomes evolving with either positive or negative selection bias. We have performed a codon-based test to identify selection pressure in genomes using the Nei-Gojobori method. The probability of rejecting the null hypothesis of strict-neutrality (dN = dS) in favour of the alternative hypothesis (dN < dS for purifying selection and dN > dS for positive selection) is shown in Figure 4.  Figure 4(a) reveals viral genomes evolve under the influence of purifying selection. Four viral genomes from the USA and another one from Hong Kong, China, is under purifying selection pressure. On the other hand, nine SARS-CoV2 genomes show strong evidence of positive selections during the outbreak (Figure 4(b)). Viral genomes from Brazil and Australia, two genomes from India, four viral genomes reported from the USA, and a viral genome from Guangdong, China, are evolving with strong positive selection bias where the rate of nonsynonymous mutations is significantly higher.

We have further analysed the genomes with positive and negative selection bias separately for the identifications of sites under strong selection biases. This segregation process helps us to identify strongly selected sites in those viral strains evolving under strong overall selection pressure and distinct mutational bias compared to the rest of the circulating viral strains. During the early pandemic period, the rate of mutations is high which is strongly influenced by the genetic diversity of the exposed host population. The synonymous mutations are much higher than the nonsynonymous mutations. All the mutations are not significant, and mutations of the naturally selected viral strains only persist. Thus, the genome segregation based on the overall selection pressure ensures the identification of significantly selected sites. In the two datasets, we have included the viral genome from Wuhan, China, as a reference genome of origin and another sequence from USA (GI no: 1820552687; accession no: MT188341.1) which shows a higher degree of evolutionary divergence (Figure 2(a)). The SLAC (single likelihood ancestor counting) method has been used to infer the ancestral state using the maximum likelihood method and also elucidates the dS and dN values and their differences at each site.

**Figure 5:**
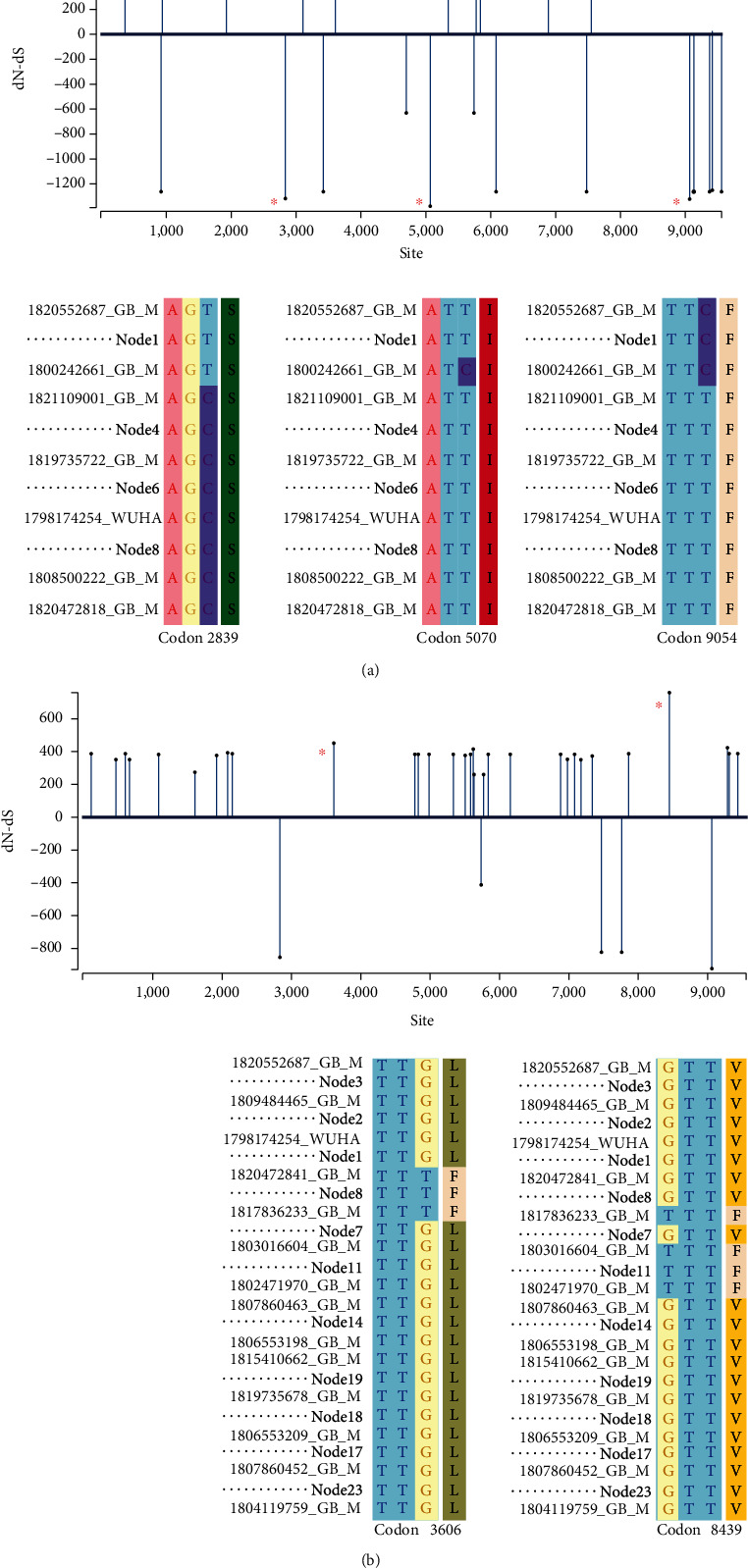
(a) Selection site analysis of SARS-CoV2 genomes under the influence of purifying selection (upper panel) and corresponding alignment at the particular codon position under high purifying selection bias (indicated by ∗) is shown in the lower panel. (b) Selection site analysis of SARS-CoV2 genomes under the positive selection bias (upper panel) and the corresponding alignment at the particular codon position (indicated by ∗) under high positive selection bias is shown in the lower panel.

SARS-CoV2 genomes with purifying selection evolved with strong biases, as the calculated dN/dS of 0.42 which is much lower than 1. Site analysis clearly shows that more sites under strong negative selection bias in comparison to the number of sites with positive selection bias (Figure 5(a)). Three codon positions, namely 2839 on nsp3, 5070 at RNA-dependent RNA polymerase (nsp12), and 9054 on the M protein are under strong purifying selection bias. At the 2839 position, there is a change of nucleotide from C to T, while for the other two sites, we have noticed substitution from T to C (Figure 4(a), lower panel). Notably, the rate of substitution calculation reveals the highest transition rate from C to T or vice versa among all possible substitution rates. Site analysis of SARS-CoV2 genomes under strong positive selection bias is shown in Figure 5(b). The calculated dN/dS is 3.03, which signifies a strong positive selection bias on those viral strains. The number of sites under strong positive selection bias is significantly higher than the number of sites with negative selection bias (Figure 5(b)). Numbers of positively selected sites are distributed through the genome. However, two sites are under strong selection bias. Codon position at 3606 on 3C-like proteinase is under strong positive selection bias where a change in the 3rd codon from G to T leads to a change in amino acid from leucine to phenylalanine. Both of them are hydrophobic amino acid with comparable size; however, the presence of aromatic ring in phenylalanine makes it more suitable interaction site. Thus, this nonsynonymous mutation might have a functional role. On the other hand, the site at 8439 codon position on surface glycoprotein is also under strong positive selection bias where a nonsynonymous substitution from G to T at the 1st codon position leads to a change in amino acid from valine to phenylalanine which is also a similar gain in function.

## 4. Conclusion

Here, we have carried out a detailed evolutionary analysis on 82 SARS-CoV2 genomes obtained from different demographic regions of the world. We have observed that even in this short evolutionary timescale, there is emerging genetic diversity across the viral population. Particularly, few genomes are evolving with a higher mutational rate with a distinct signature of nucleotide substitution. We found four viral genomes are under the effect of purifying selection, while nine SARS-CoV2 genomes that include genomes from Brazil, Australia, India, and the USA are under strong positive selection bias. Site analysis indicates that two sites at 3606 and 8439 on 3C-like proteinase and spike protein, respectively, are evolving under strong positive selection bias where a hydrophobic amino acid changes to phenylalanine which makes these sites capable of interacting strongly with interaction partners, implying a gain in function. Our study elucidates adaptation of few SARS-CoV2 viral strains under the genomic compositional constraints during the outbreak shaped by the natural selection.

## Data Availability

The dataset used for this study is available upon request.

## Supplementary Materials

Supplementary Materials Table S1: description of the 82 SARS-CoV2 genomes obtained from the NCBI Genome database is provided in a tabular form.
