# Supplementary material for: Phylogenomics Analysis of SARS-CoV2 Genomes Reveals Distinct Selection Pressure on Different Viral Strains
Source: Biomed Res Int. 2020 Nov 27;2020:5746461. doi: 10.1155/2020/5746461 (PMC7703455; doi:10.1155/2020/5746461)
Supplement: Supplementary Materials — Table S1: description of the 82 SARS-CoV2 genomes obtained from the NCBI Genome database is provided in a tabular form. [file 5746461.f1.docx]

**Supporting information**

**Phylogenomics analysis of SARS-CoV2 genomes reveals distinct selection pressure on different viral strains**

Sanjana Ghosh, Sandipan Chakraborty*

**Amity Institute of Biotechnology, Amity University, Kolkata 700135, India**

*Corresponding author:

Sandipan Chakraborty:

E-mail: [sandipanchakraborty.13@gmail.com](mailto:sandipanchakraborty.13@gmail.com); [schakraborty8@kol.amity.edu](mailto:schakraborty8@kol.amity.edu)

**Table S1:** List of the 82 SARS-CoV2 genomes obtained from the NCBI Genome database.

| Genome index | Gi number | Accession number | Country | State | Coding region |
| --- | --- | --- | --- | --- | --- |
| 1 | 1820552687 | MT188341.1 | USA | Minnesota | 212-29623 |
| 2 | 1820552665 | MT188339.1 | USA | Minnesota | 212-29620 |
| 3 | 1820097838 | MT163719.1 | USA | Washington | 266-29674 |
| 4 | 1818798657 | MT152824.1 | USA | Snohomish County- WA | 264-29672 |
| 5 | 1820097825 | MT163718.1 | USA | Washington | 266-29674 |
| 6 | 1820097812 | MT163717.1 | USA | Washington | 260-29668 |
| 7 | 1802471959 | MN994467.1 | USA | California | 266-29674 |
| 8 | 1808500211 | MT044257.1 | USA | Illinois | 266-29674 |
| 9 | 1812103020 | MT106054.1 | USA | Texas | 266-29674 |
| 10 | 1809484465 | MT050493.1 | IND | Kerala | 246-29654 |
| 11 | 1818244605 | MT135042.1 | CHN | Beijing | 266-29674 |
| 12 | 1818244627 | MT135044.1 | CHN | Beijing | 266-29674 |
| 13 | 1818244594 | MT135041.1 | CHN | Beijing | 266-29674 |
| 14 | 1818244616 | MT135043.1 | CHN | Beijing | 266-29674 |
| 15 | 1800242661 | MN975262.1 | CHN | Hong Kong-Shenzhen | 266-29674 |
| 16 | 1820518901 | MT123292.2 | CHN | Guangzhou | 266-29674 |
| 17 | 1808633715 | MT049951.1 | CHN | Yunnan | 266-29674 |
| 18 | 1802498786 | MN997409.1 | USA | Arizona | 266-29674 |
| 19 | 1812102998 | MT106052.1 | USA | California | 266-29674 |
| 20 | 1800408777 | MN985325.1 | USA | Washington | 266-29674 |
| 21 | 1805599865 | MT020881.1 | USA | Washington | 266-29674 |
| 22 | 1805599854 | MT020880.1 | USA | Washington | 266-29674 |
| 23 | 1809484493 | MT066175.1 | Taiwan |  | 266-29674 |
| 24 | 1804119759 | MT012098.1 | IND | Kerala | 253-29658 |
| 25 | 1807860439 | MT039890.1 | South Korea |  | 266-29674 |
| 26 | 1820472818 | MT184911.1 | USA | Cruise | 266-29674 |
| 27 | 1800489756 | MN988713.1 | USA | Illinois (IL) | 266-29674 |
| 28 | 1820518900 | MT123291.2 | CHN | Guangzhou | 263-29671 |
| 29 | 1820518902 | MT123293.2 | CHN | Guangzhou | 260-29668 |
| 30 | 1811294619 | MT093571.1 | Sweden |  | 266-29674 |
| 31 | 1808500222 | MT044258.1 | USA | California | 266-29650 |
| 32 | 1819735678 | MT159716.1 | USA | Cruise | 266-29659 |
| 33 | 1807860452 | MT039887.1 | USA | Wisconsin | 266-29671 |
| 34 | 1820472841 | MT184913.1 | USA | Cruise | 266-29674 |
| 35 | 1821109001 | MT192765.1 | USA | San Diego County | 266-29674 |
| 36 | 1820097799 | MT163716.1 | USA | Washington | 266-29674 |
| 37 | 1807860463 | MT039888.1 | USA | Massachusetts | 266-29674 |
| 38 | 1819735744 | MT159722.1 | USA | Cruise | 266-29674 |
| 39 | 1819735443 | MT159705.1 | USA | Cruise | 266-29674 |
| 40 | 1806553198 | MT027063.1 | USA | California | 266-29674 |
| 41 | 1806553187 | MT027062.1 | USA | California | 266-29674 |
| 42 | 1820472807 | MT184910.1 | USA | Cruise | 266-29674 |
| 43 | 1809484476 | MT066156.1 | Italy |  | 266-29674 |
| 44 | 1819735634 | MT159712.1 | USA | Cruise | 266-29674 |
| 45 | 1817836233 | MT126808.1 | Brazil |  | 266-29674 |
| 46 | 1805293611 | MT019529.1 | China | Hubei, Wuhan | 266-29674 |
| 47 | 1803016604 | MT007544.1 | Australia | Victoria | 266-29674 |
| 48 | 1820552676 | MT188340.1 | USA | MN | 212-29620 |
| 49 | 1820472785 | MT184908.1 | USA | Cruise | 266-29674 |
| 50 | 1819735722 | MT159720.1 | USA | Cruise | 266-29674 |
| 51 | 1819735689 | MT159717.1 | USA | Cruise | 266-29674 |
| 52 | 1815410662 | MT123290.1 | CHN | Guangdong, Guangzhou | 269-29677 |
| 53 | 1809484504 | MT066176.1 | Taiwan |  | 266-29674 |
| 54 | 1806553209 | MT027064.1 | USA | California | 266-29674 |
| 55 | 1802471970 | MN994468.1 | USA | California | 266-29674 |
| 56 | 1821109024 | MT192772.1 | Viet Nam | Ho Chi Minh city | 266-29674 |
| 57 | 1821109035 | MT192773.1 | Viet Nam | Ho Chi Minh city | 265-29673 |
| 58 | 1820472829 | MT184912.1 | USA | Cruise | 266-29674 |
| 59 | 1819735484 | MT159707.1 | USA | Cruise | 266-29674 |
| 60 | 1819735567 | MT159709.1 | USA | Cruise | 266-29674 |
| 61 | 1819735667 | MT159715.1 | USA | Cruise | 266-29674 |
| 62 | 1819735506 | MT159708.1 | USA | Cruise | 266-29674 |
| 63 | 1819735700 | MT159718.1 | USA | Cruise | 266-29674 |
| 64 | 1819735473 | MT159706.1 | USA | Cruise | 266-29674 |
| 65 | 1812779165 | MT118835.1 | USA | Cruise | 266-29674 |
| 66 | 1812103009 | MT106053.1 | USA | Cruise | 266-29674 |
| 67 | 1810678290 | MT072688.1 | Nepal |  | 251-29659 |
| 68 | 1805293633 | MT019531.1 | China | Hubei, Wuhan | 266-29674 |
| 69 | 1798174254 |  | China | Wuhan | 266-29674 |
| 70 | 1805293644 | MT019532.1 | China | Hubei, Wuhan | 266-29674 |
| 71 | 1805293622 | MT019530.1 | China | Wuhan | 266-29764 |
| 72 | 1807860410 | MT039873.1 | China | Hangzhou | 263-29671 |
| 73 | 1819735656 | MT159714.1 | USA | Cruise | 266-29674 |
| 74 | 1819735711 | MT159719.1 | USA | Cruise | 266-29674 |
| 75 | 1819735645 | MT159713.1 | USA | Cruise | 266-29674 |
| 76 | 1819735623 | MT159711.1 | USA | Cruise | 266-29674 |
| 77 | 1819735733 | MT159721.1 | USA | Cruise | 266-29674 |
| 78 | 1819735612 | MT159710.1 | USA | Cruise | 266-29674 |
| 79 | 1820472796 | MT184909.1 | USA | Cruise | 266-29674 |
| 80 | 1820472774 | MT184907.1 | USA | Cruise | 266-29674 |
| 81 | 1821108987 | MT192759.1 | Taiwan |  | 239-29647 |
| 82 | 1805293655 | MT019533.1 | China | Hubei, Wuhan | 266-29674 |
